# Study on Immune Response of Organs of *Epinephelus coioides* and *Carassius auratus* After Immersion Vaccination With Inactivated *Vibrio harveyi* Vaccine

**DOI:** 10.3389/fimmu.2020.622387

**Published:** 2021-02-09

**Authors:** Hua Gong, Qing Wang, Yingtiao Lai, Changchen Zhao, Chenwen Sun, Zonghui Chen, Jiafa Tao, Zhibin Huang

**Affiliations:** Key Lab of Aquatic Animal Immune Technology of Guangdong Province, Key Lab of Fishery Drug Development of Ministry of Agriculture and Rural Affairs, Pearl River Fisheries Research Institute of Chinese Academy of Fishery Sciences, Guangzhou, China

**Keywords:** immersion vaccination, mucosal immunity, immune mechanism, immune synergy of hindgut-liver-spleen, IgM, MHC II, Pearson correlation coefficient(*r*)

## Abstract

Immersion vaccination relies on the response of fish mucosa-associated lymphoid tissues, the Crucian carp (*Carassius auratus*) and Grouper (*Epinephelus coioides*) were researched in this paper to examine local mucosal immune responses and associated humoral system responses following immersion vaccination. We administered 1.5 × 10^7^ CFU/ml formalin-inactivated *Vibrio harveyi* cells and measured mucus and serum antibody titers as well as IgM, MHC II mRNA levels in immune organs. The mucosal antibody response preceded the serum response indicating a role for local mucosal immunity in immersion vaccination. IgM and MHC II mRNA levels were relatively greater for the spleen and head kidney indicating the importance and central position of systemic immunity. Expression levels were also high for the gills while skin levels were the lowest. IgM and MHC II mRNA levels were altered over time following vaccination and the hindgut, liver and spleen were similar indicating a close relationship, so the absolute value of *r* is used to analyze the correlation among different organs immunized. It can be inferred the existence of an internal immune molecular mechanism for Immune synergy hindgut-liver-spleen, from the peak time (14^th^ day), the relative ratio of genes expression in the same tissues between the immunized grouper and the control group (26 times), and Pearson correlation coefficient (0.8<|*r*|<1). Injection challenges with live *V. harveyi* indicated that the relative protection rates for the crucian carp and Grouper was basically the same at 44.4% and 47.4%, respectively. It is believe that crucian carp may be used as a substitute for the valuable grouper in immunity experiment, just from aspect of the relative percent survival (RPS) and how it changes with time. But they were not consistent about the IgM mRNA expression between that of crucian carp and grouper after immersion the *Vibrio* vaccine.

## Introduction

Immersion vaccination has numerous advantages over traditional methods including minimal pain for the fish, lower labor costs, and time coupled with greater operator safety especially for large numbers of small fish. There are numerous commercial products used for this type of procedure that are currently in use in Europe, America, and East Asia and is the recommended immunization procedure (1, 2). However, immersion vaccination also possesses drawbacks such as the generation of weak immune responses and a large amount of vaccine is necessary that can increase costs (3). Therefore, it is of great theoretical significance and practical value to systematically study the mechanism of immersion vaccination and its influencing factors in order to develop efficient, stable, inexpensive, and practical vaccines (2, 4).

There are many aspects of fish immunology still unknown and we are far from close to understanding on which immune mechanisms the protection against many of these pathogens resides (5). Mucosal surfaces of fish, including skin, gill, and gut, contain numerous immune substances poorly studied that act as the first line of defense against a broad spectrum of pathogens (6), and these organs provide the local immune responses required for a competent immune system in fish. Among the containing immune substances, Immunoglobulin M (IgM) is the first antibody that is produced in the immune system and provides a crucial first line of defense for the immune system (7). So far, three main Ig isotypes have been identified in teleosts, including IgM, IgD, and IgT/Z (8, 9), tetrameric IgM is widely accepted as the prevalent serum and mucus Ig type in most teleosts (3, 6). And IgT/Z is thought to be specialized in mucosal immunity, but as known that there is not every teleost species possesses these isotypes Ig (3, 9). This suggested that IgM was the most important mediator in the fish specific humoral and mucosal immune responses (3, 8–10).

The genes of the MHC are recognized as an essential component of the vertebrate adaptive immune system, and are responsible for the recognition and presentation of foreign antigens (11). Classical MHC class IImolecules (MHC II) are restricted to professional antigen presenting cells, which activates B cell differentiation into plasma cells, producing antibodies specific to the invading pathogen, and memory cells, preserving a record of past infection (12, 13). MHC II-dependent immune memory is considered an essential component of the adaptive immune response (14).

In the current study, we utilized immersion vaccination of inactivated *Vibrio harveyi* cells for the Crucian carp (*Carassius auratus*) and Grouper (*Epinephelus coioides*) and measured alterations in antibody titers in skin mucus and serum and IgM mRNA levels in gill, skin, hindgut, liver, spleen, and head kidney (HK). We also challenged the fish with live *V. harveyi* by injection to determine the protective effect of vaccination over time. The mucosal and systemic immune responses that we measured will provide theoretical support for research and development as well as for the practical use of fish immersion vaccines.

## Materials and Methods

### Materials

Crucian carp and grouper (400 each) from the Guangdong Daya Bay Fisheries Experimental Center and a farm in Nanhai, Guangdong Province that possessed average lengths of 10 ± 1.5 cm. The fish were acclimated for 7 days and randomly divided into two groups: 200 were used for immunization and 100 were retained as controls. The water temperature was maintained at 28 ± 2.0°C. The bacterial pathogen *V. harveyi* strain SpGY020601 was isolated and identified at Aquatic Diseases and Immunity Laboratory of the Pearl River Fisheries Research Institute (15–17). The bacteria were inactivated by exposure to 0.3% formalin as previously reported (18). The immersion vaccination protocol utilized 1.5 × 107 CFU/ml and a 30-min immersion time in 0.65% normal saline for crucian carp and 0.85% for grouper. Control fish received immersion in saline in the absence of the inactivated bacteria.

### Indirect ELISA

#### Serum and Skin Mucus Collection

Serum and mucosal samples were taken from 9 fish at random on days 2, 4, 7, 11, 14, 21, and 28 after immunization. The skin surfaces were lightly scraped with clean glass slides and resulting mucus of three fish per sample time and group was mixed. An equal volume of 0.85% normal saline was then added and the solution was centrifuged at 10,000 rpm for 20 min and the supernatant was collected. Blood samples (0.3 ml) was sampled from the tail vein and allowed to stand at room temperature for 1 h and then incubated overnight at 4°C. The samples were then and centrifuged at 4,500 rpm for 15 min and the serum was collected and used for analysis.

#### Determination of Antibody Titer

IgM antibody titers were determined as previously described (10, 19, 20). In brief, 96-well ELISA plates were coated with formalin-inactivated *V. harveyi* suspensions overnight at 4°C and then blocked using 5% skimmed milk at 37°C for 1.5 h. Diluted mucus samples were then added followed by incubation at 37°C for 1.5 h. The antibodies used for the ELISA were mouse anti-grouper IgM monoclonal Mab-2D3 (Institute of Animal Husbandry and Veterinary of Fujian Academy of Agricultural Sciences, China) (21) and a horseradish peroxidase-labeled sheep anti-mouse IgG (Jackson, USA).Tetramethylbenzidine was then added and the reactions were terminated with the addition of 2 M sulfuric acid after 20 min. A negative control using serum or mucus from non-immunized fish and blank controls containing only the suspending solutions were used at the same time. Absorbance was measured at 450 nm using a Multiskan Mk3 instrument (Thermo Fisher, Pittsburg, PA, USA). Samples were judged positive when the optical density of the samples was ≥ 2.1 than the blank control.

### Real-Time PCR

The primer sequences used for detection of immune gene expression are listed in **Table 1**. And the primers used for gene detection were designed using Primer Express 3.0 software (Thermo Fisher) for the grouper IgM, MHC II and internal reference 18S rDNA and for the crucian carp IgM and internal reference β-actin. Specificity of the primers was tested using NCBI BLAST (https://blast.ncbi.nlm.nih.gov/Blast). Primers were synthesized by Shanghai Bioengineering Technology (Shanghai, China).

**Table 1:** Primers used for RT-PCR.

Fish	Gene	Primer Sequence	Product (bp)
Crucian carp (*Carassius auratus*)	IgM	F 5′- TGACCCTGACTTGCTATGTG-3′ R 5′-CAGAGACTGGTGGTGAACTAC-3′	218
	β actin	F 5′-AGAGGGAAATCGTGCGTG-3′ R 5′-GAAGGAAGGCTGGAAGAGG-3′	185
Grouper (*Epinephelus coioides*)	IgM	F 5′-GCCTGTATCCTGATTGTCGG-3′ R 5′-GCTGGGACTTCAGGTTGTTG-3′	115
	MHC II	F 5′-GGACATCAGACCCTGGACCAA-3′ R 5′-ACACCGAGCAGACCGACAGT-3′	105
	18s DNA	F 5′-GGACACGGAAAGGATTGACAG-3′ R 5′– CGGAGTCTCGTTCGTTATCGG-3′	99

And the PCR reaction system(25 μl) as following:

$$SYBR\;Premix\;Ex\;Taq^{™}\left(2\times \right)\;12.5\;\mu l\;\text{Primer}\;\text{F}/\text{R}\;\left(10\;\mu \text{mol}/L\;each\right)\;0.5\;\mu l$$

RT 1 μl Nuclease-free water  10.5 μl

Total RNA was extracted from gill, skin, hindgut, liver, spleen and HK using Trizol reagent (Invitrogen, Carlsbad, CA, USA). RNA levels were quantified using UV spectroscopy and OD_260_/OD_280_ ratios between 1.8 and 2.0 were considered sufficient for analysis. Electrophoresis in 1% agarose gels was used to identify the integrity of the extracted RNA. RNA was reverse-transcribed using 1 μg RNA in a 20-μl total volume using a M-MLV reverse transcription kit according to the manufacturer’s instructions (Promega, Madison, WI, USA).

Real-time qPCR analysis was performed using 2 μl cDNA template, 10.4 μl of SYBR premix ex Taq (Takara, Japan), 0.4 μl of 10 μM primers and 6.8 μl of deionized water. Amplification conditions using an Real-time fluorescence quantitative PCR instrument (ABI7500, USA) were as follows: 95°C for 4 min, followed by 35 cycles of 95°C for 20 s, 56°C for 20 s, and 72°C for 20 s and a final step of 72°C for 5 min. The integrity of the amplified products was assessed using melting curve analysis using the software supplied with the instrument.

### Demonstration of Protective Immunity

The bacterial suspension (1.8 × 10^9^ CFU/ml) was diluted five times continuously with sterile physiological saline, then the crucian carp and grouper were injected intraperitoneally with 5^0^ to 5^−4^ dilution bacterial suspension at a dose of 0.2 ml/tail. The control group was injected with the same amount of sterile saline. During the test, the water temperature was 28 ± 1°C. The fish were observed continuously for one week, the death number of experimental animals was recorded, and the median lethal dose (LD50) was calculated according to Reed&Muench method (Reed L J & Muench, 1938).

The protective function of the *V. harveyi* vaccination was examined using 20 fish from each group at 1, 2, 3, 4, and 5 weeks following vaccination. The fish were randomly selected and received 0.2 ml of 1.2 × 10^8^ CFU/ml of *V. harveyi* by intraperitoneal injection. The fish were maintained for 2 weeks with a tank temperature of 28 ± 1°C and monitored for signs of morbidity and mortality. The relative percent survival (RPS) was calculated according to the following formula:

$$RPS=\left(1-\frac{\text{mortality}\;\text{rate}\;\text{of}\;\text{immune}\;\text{group}}{\text{mortality}\;\text{rate}\;\text{of}\;\text{control}\;\text{group}}\right)\times 100\%$$

### Data Analysis

The experimental data were expressed as mean ± SD and levels of significance were assessed using one-way ANOVA using SPSS14 (IBM, Chicago, IL, USA), the LSD method for the Student’s *t*-test, and the correlation analysis of different organs of grouper after immersion with inactivated *V. harveyi* vaccine was using Pearson correlation coefficient(*r*), which calculated according to the following formula (22):

$$r=\frac{\Sigma \left(x-\bar{x}\right)\left(y-\bar{y}\right)}{\sqrt{\Sigma {\left(x-\bar{x}\right)}^{2}\Sigma {\left(y-\bar{y}\right)}^{2}}}$$

The absolute value of *r* is used to analyze the correlation, the larger the value of |r|, the closer the relationship is. Among them, |*r*|<0.3, means no correlation; 0.3≤|*r*|<0.5, means low linear correlation; 0.5≤|*r*|<0.8, means moderately linear correlation; 0.8≤|*r*|≤1, means highly linear correlation.

## Results

### Dynamic Changes of Mucus and Serum Antibody Titers

**Figure 1:**
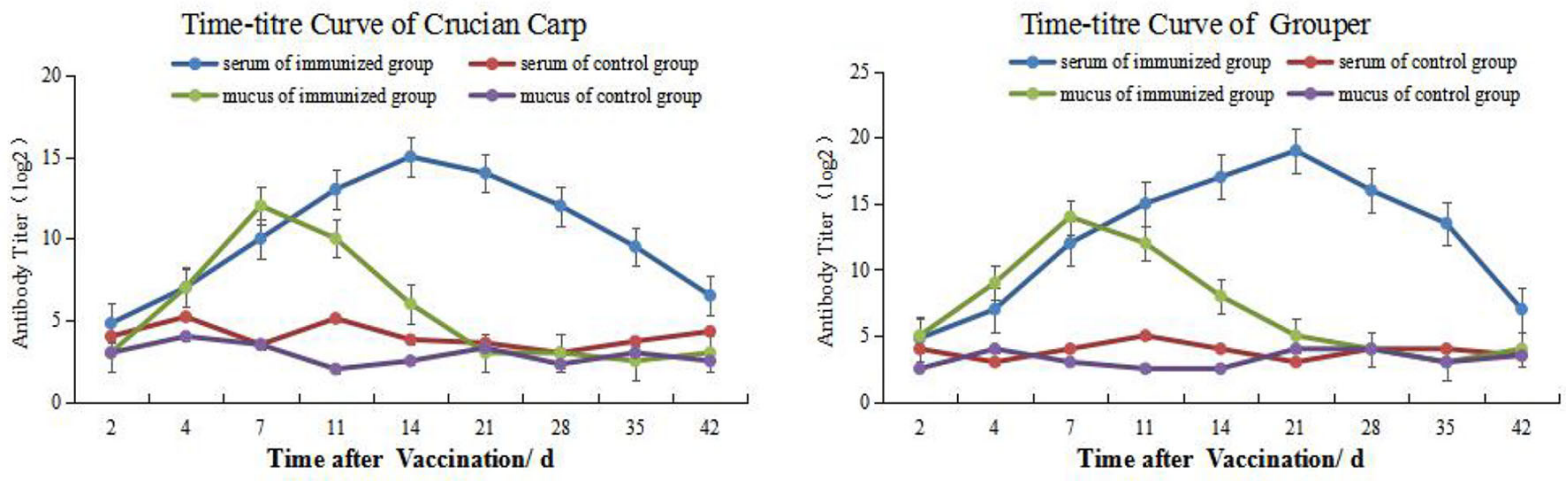
Time-and antibody titers of *Carassius auratus* (left) and *Epinephelus coioides* (right).

We used an indirect ELISA method to follow alterations in antibody titers of mucus and blood over time. The antibody titers in carp skin mucus peaked on day 7 and then declined and returned to pre-immunization levels by week 3. The titers in the control group were not significantly altered during the whole time course. Overall, except for days 0 (the day of the vaccination) and 21, antibody titers in the immune groups were significantly higher than for the controls (*P* < 0.05). On day 21 the serum titers peaked and then declined but remained at high level to weeks 6 where immune group titers were significantly higher than controls (*P* < 0.05) (**Figure 1**, left). The titers for the grouper showed similar changes and mucus titers peaked on day 7 while serum titers were maximal on day 21 (**Figure 1**, right).

### Alterations in IgM mRNA Expression in Different Tissues

RNA samples taken from the HK, spleen, liver, hindgut, gills and skin were initially examined by gel electrophoresis to determine RNA integrity. These samples contained sharp bands corresponding to 28S and 18S rRNA indicating the absence of degradation in the purification procedure (**Figure S1**). These samples were then used of RT-PCR analysis to measure tissue levels of IgM mRNA. The mRNA of IgM mRNA and β-actin gene were used as the standard, although there was a good similarity with the samples to be tested, problems such as the dissolution medium of the standard and the extraction method of nucleic acid would affect the stability of the standard curve (23). It can be known that when SYBR Green I fluorescent dye is used for quantitative PCR detection, the formation and interference of primer dimers can also be effectively avoided by optimizing the reaction system, and specific PCR products can be obtained.

**Figure 2:**
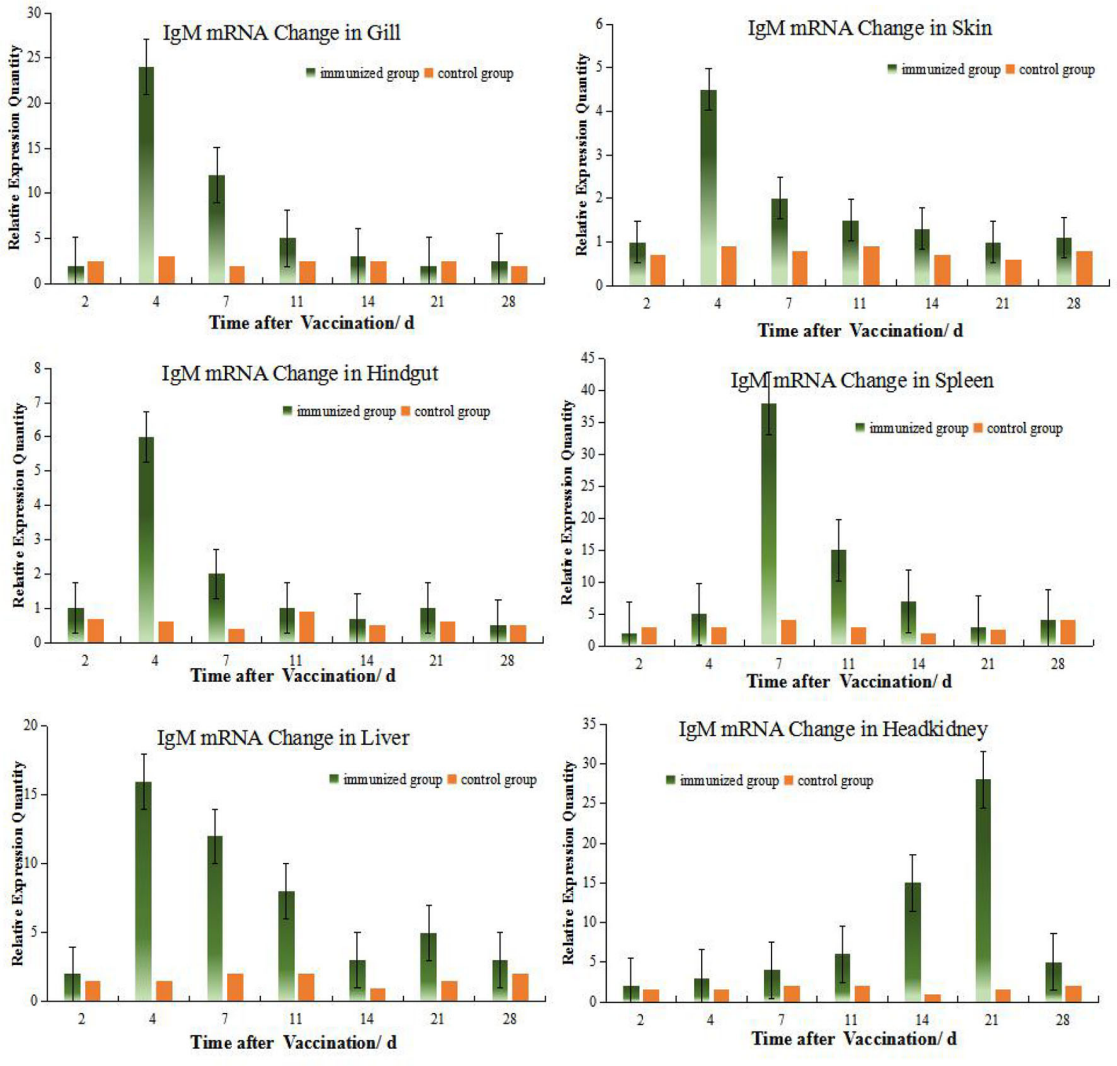
Relative quantification of crucian carp IgM gene mRNA in different organs. The relative abundance for IgM mRNA from the tissues was ranked as spleen > HK> gill >liver > hindgut>skin. The alterations in mRNA levels over time were similar for the gills, skin, hindgut and liver with increases at day 4 post-inoculation. And there were clear change difference between spleen and HK at this experiment, although both organs are central immune organs.

**Figure 3:**
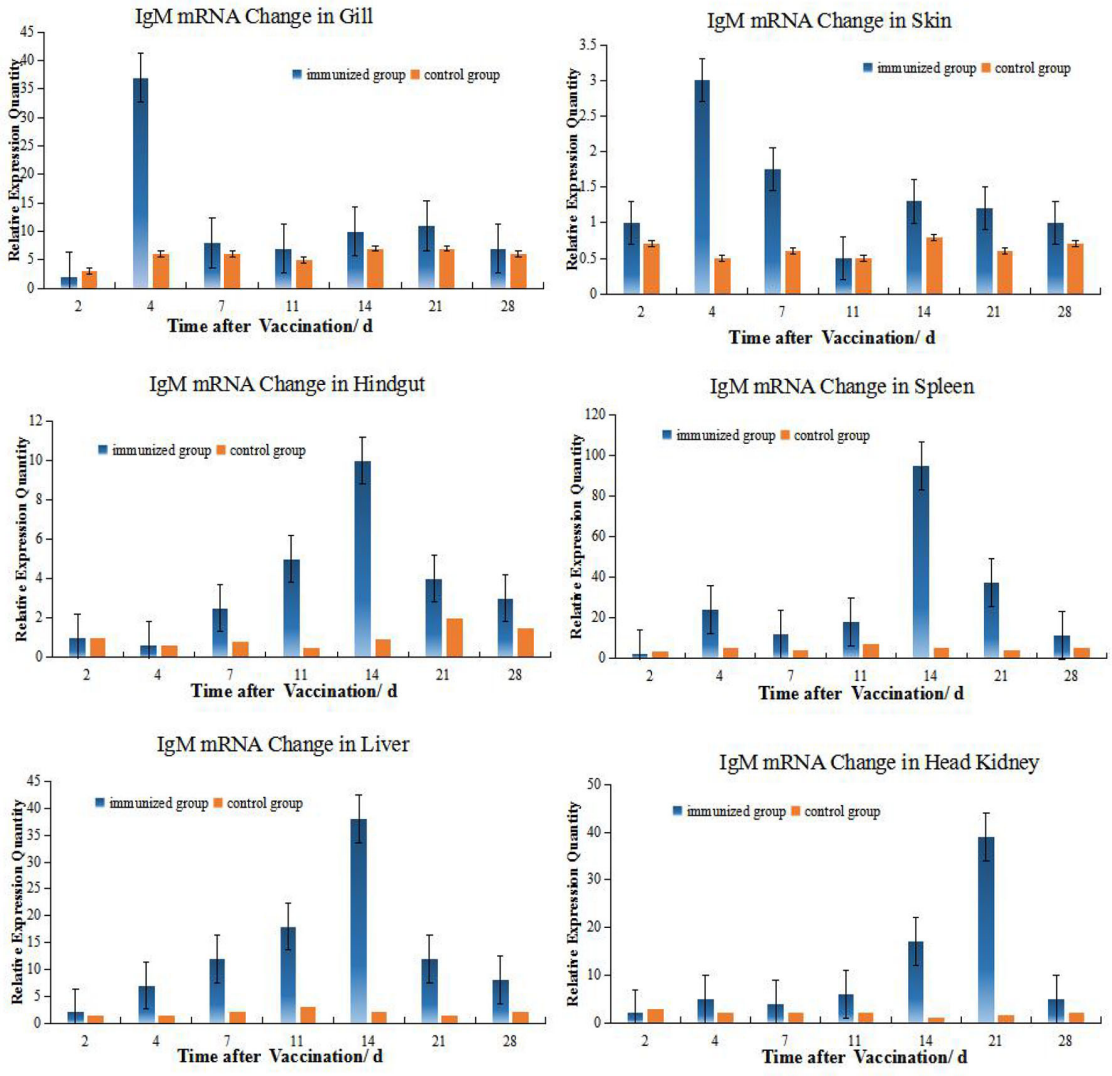
Relative quantification of grouper IgM gene mRNA in different organs. The relative abundance for IgM mRNA from the tissues was ranked as spleen > HK> liver>gill> hindgut>skin. The alterations in mRNA levels over time were similar for the hindgut, liver, and spleen with increases at day 14 post-inoculation. Organ IgM mRNA expression levels changed with time and the hindgut, liver and spleen followed the same temporal trend.

The levels of IgM mRNA were up-regulated in all six tissues after immersion vaccination. The most significant changes for both fish were seen in the spleen that were increased 93- and 36-fold for the grouper and the crucian carp respectively. The relative abundance for IgM mRNA from the crucian carp tissues was ranked as spleen > HK> liver >gill > hindgut>skin, while grouper was spleen > HK> liver >gill > hindgut>skin. The alterations in mRNA levels over time were similar between the two fish for the gills and skin with increases at day 4 post-inoculation. Expression levels in spleen increased at days 14 and 7, and in the hindgut on days 14 and 4 for the grouper and crucian carp, respectively. The up-regulation of expression in the HK appeared later and was maximal at day 21 for both fish (**Figures 2**, **3**).

### Alterations in MHC II mRNA Expression in Different Tissues of Grouper

**Figure 4:**
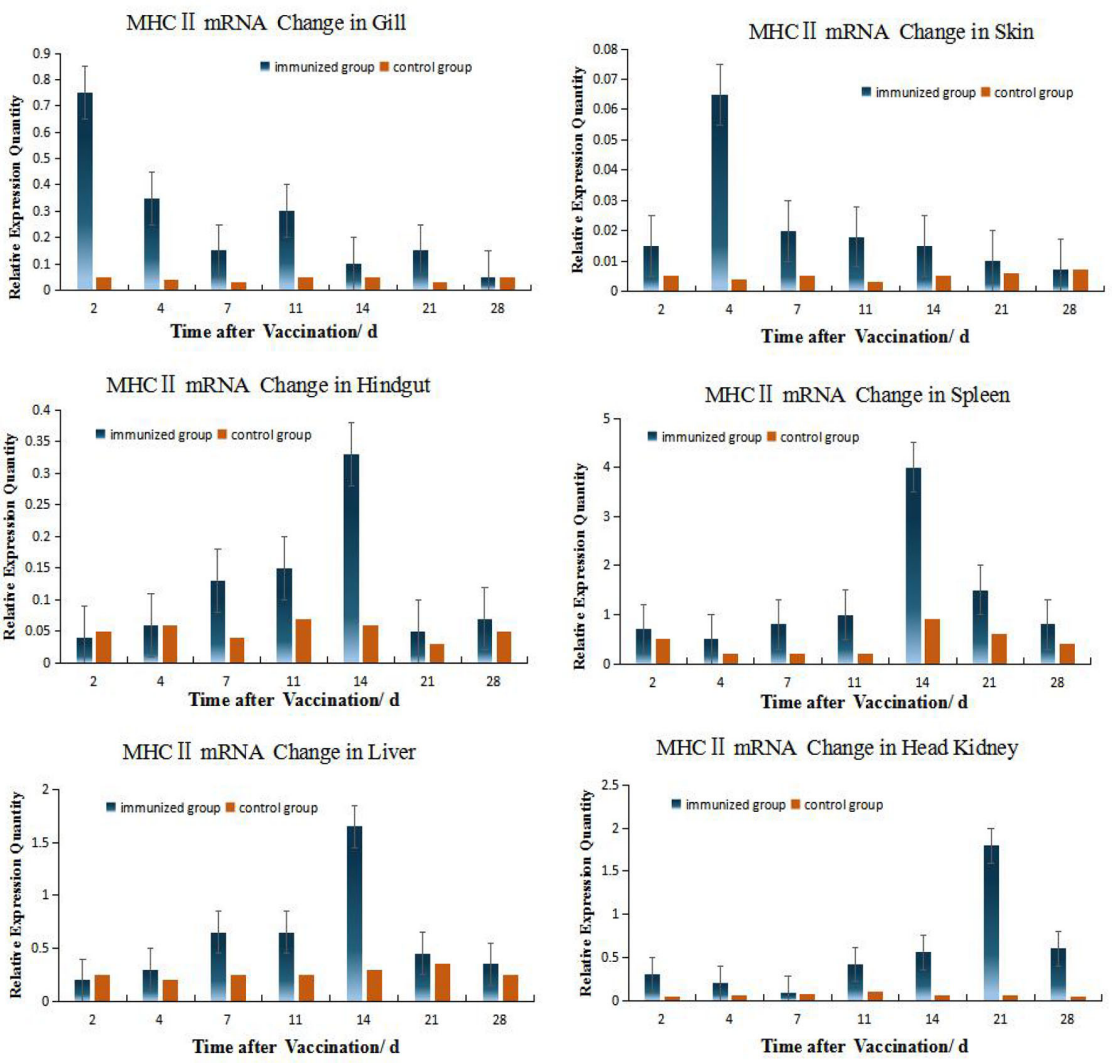
Relative quantification of grouper MHC II gene mRNA in different organs. The relative abundance for MHC II mRNA from the tissues was ranked as spleen > HK> liver>gill> hindgut>skin. The alterations in mRNA levels over time were similar for the hindgut, liver, and spleen with increases at day 14 post-inoculation. Organ MHC II mRNA expression levels changed with time and the hindgut, liver and spleen followed the same temporal trend.

According to the relative expression of MHC- II of grouper different organs (**Figure 4**), Up-regulation of MHC gene expression was detected in six organs after immersion vaccination. The relative expression level of MHC II in HK is relatively high (the peak value is close to 2.0), while the relative expression level in skin is the smallest (the peak value is only 0.064), and the expression level in gill and hindgut is relatively low. But the MHC II expressions quickly reach the peak in gill and skin, which shows the important role of gill and skin in antigen presentation and transmission during immersion (3, 5, 8). The same dynamic changes were observed in hindgut, spleen, and liver, and the expression levels were obviously increased within 4 days after immunization, and reached the peak at 14 days all. The expression of MHC II was up-regulated fastest in gill, reached its peak 2 days after immunization, while peaked up-regulated later in HK, but the up-regulation lasted for a long time until the 28th day.

### Relation of Different Organs During Immunization Reaction

Among the 6 organs of immunized grouper, the expression of MHC II was up-regulated fastest in gill, reached its peak 2 days after immunization. Besides this, the expressions of gene peaked all in the 4^th^ day in the gill and skin. And the gene expressions peak time of grouper hindgut, spleen and liver were all in 14^th^ day after vaccination, but these in crucian carp were near the 4^th^ day all, which indicated that there is difference between this two species fish in immunity protection mechanism.

The expressions of IgM and MHC II up-regulated most were all in HK, about 26 times higher than that of control group 21 days after immunization, and the level just like the IgM expression in grouper HK after *V. alginolyticus* challenge (24). The gill was next, which was 15 times higher than the control group on the second day after immunization. The skin was up-regulated by 13 times on the 4^th^ day after immunization compared with the control group. After that, the expression level in hindgut, liver and spleen increased slightly, and they were about 6 times all higher than those in the control group on the 14th day after immunization (**Table 2**). And the same value occurred in immunized crucian carp hindgut, liver and spleen higher than the control, which were about 10 times.

**Table 2:** Statistics on the peak time and maximum value of gene expression up-regulation multiple in different tissues after immunization compared with control group.

Tissue	Gill		Skin		Hindgut		Spleen		Liver		HK	
	Peak time/d	Relative values	Peak time/d	Relative values	Peak time/d	Relative values	Peak time/d	Relative values	Peak time/d	Relative values	Peak time/d	†Relative values
Crucian Carp IgM	4	8.0	4	5.0	4	10	4	10.7	7	9.5	21	18.7
Grouper IgM	4	6.2	4	6.0	14	11.1	14	19.0	14	19.0	21	26.0
Grouper MHC II	2	15.0	4	13.0	14	5.5	14	6.0	14	5.5	21	25.7

† Relative value the ratio of gene expression in the same tissues between the immunized grouper and the control group.

**Figure 5:**
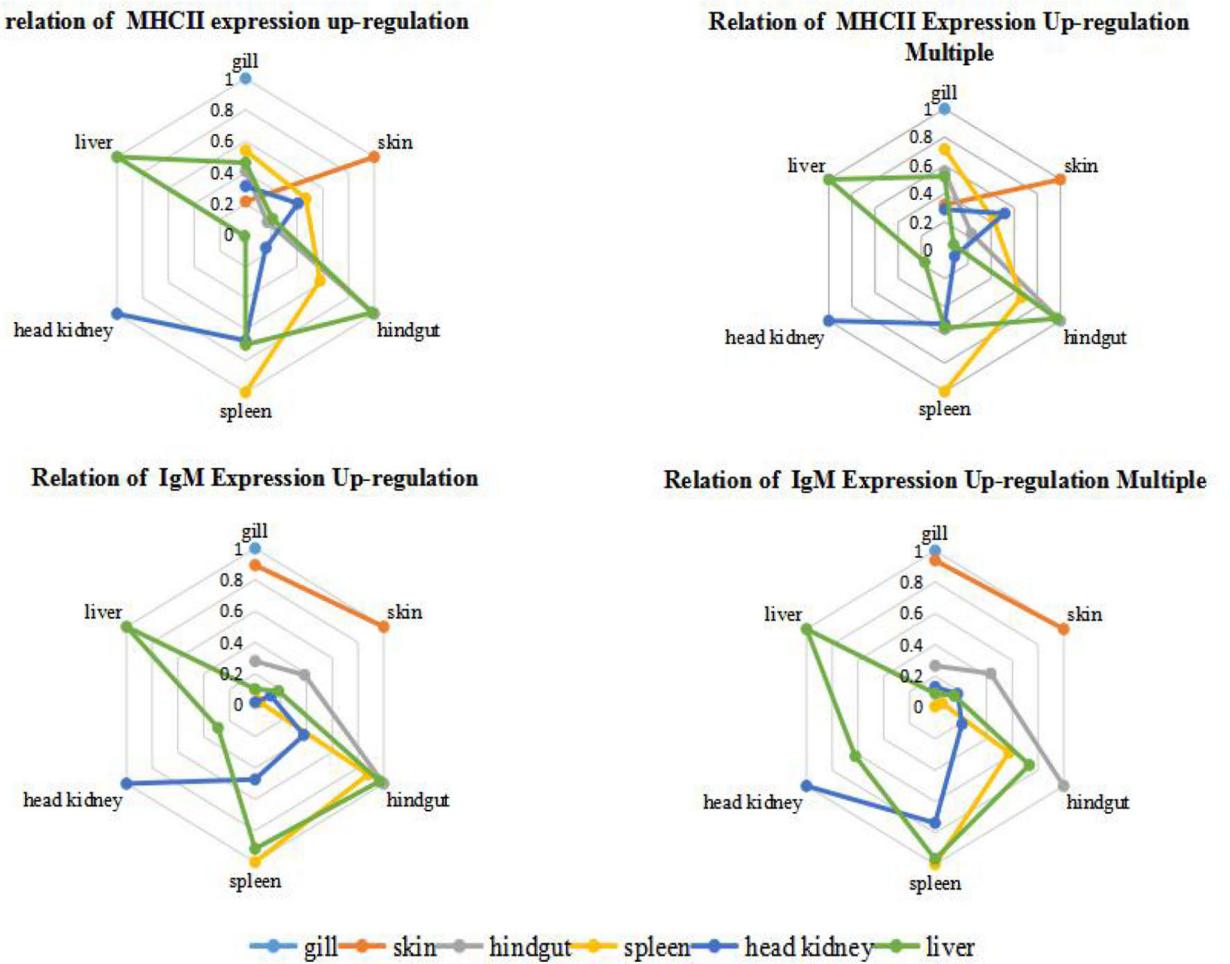
Correlation analysis of grouper organs after immersion with inactivated *V. harveyi* vaccine. The relation of gene expression up-regulation was analyzed by the absolute value of *r* between the quantitative changes of gene expression in different organs after immunization; while the relation of gene expression up-regulation multiple was analyzed by the absolute *r* value of between the relative ratio of gene expression in different organs after immunization is analyzed, and this relative ratio is the ratio of gene expression in the same tissues between the immunized grouper and the control group.

In order to quantify the comparison results, we draw lessons from the water ecological treatment method (22), and introduce Parson correlation coefficient to study the relationship between organs in immune response. Among the six organs of immunized grouper, it was found that hindgut, spleen and liver had relatively higher *r* value each other, whether in the up-regulation expression of IgM or MHC II (**Figure 5**). And the mucosal organs, such as gill and skin, had the relatively higher correlations each other in the IgM experiment, had the relatively lower correlations in the MHC II expression, showed the difference of antigen presentation about the two organs (18).

### Protective Immunity

The Results of challenge tests (**Table 3**) showed that the strain has strong toxicity to crucian carp and grouper as the reference (15, 16), the LD50 of the strain to this two species were 6.1 × 10^6^ CFU/ml both calculated by R-M method. So *V. harveyi* strain can be used with same concentration for two species of fish in the challenge protection test. To demonstrate the effectiveness of the immersion vaccination, we challenged the fish by injection of inactivated *V. harveyi* cells at weeks 1, 2, 3, 4, and 5 post–vaccination (**Table 4**). The protection after 1 week immunization was < 30% and maximal rates of 44.4% and 47.4% were achieved by week 4. The serum antibody titers for both fish mirrored these changes but the mucosal titers did not. In addition, the maximal protection against challenge appeared on week 4 (28 days) whereas the highest serum antibody levels were found on week 3 (21 days).

**Table 3:** Challenge of no-immunized fish with different concentrations of suspension *V. harveyi* administered by injection (number of deaths/number of trials).

Concentrations of suspension (CFU/ml)	1.8×10^9^	3.6×10^8^	7.2×10^7^	1.44×10^7^	2.88×10^6^	Sterile physiological saline
Crucian carp (*Carassius auratus*)	9/10	7/10	6/10	4/10	2/10	0/10
Grouper (*Epinephelus coioides*)	9/10	7/10	6/10	4/10	2/10	0/10

**Table 4:** Challenge of immunized fish with *V. harveyi* administered by injection.

Fish	Time of challenge: post-innoculation (week)	Number of experimental fish (tail)	Number of deaths (tail)	Mortality (%)	†RPS (%)
Crucian carp (*Carassius auratus*)	1 2 3 4 5 6 control	20 20 20 20 20 20 20	16 14 11 10 13 15 18	80 70 55 50 65 75 90	11.2 22.2 38.8 44.4 37.8 16.7 ——
Grouper (*Epinephelus coioides*)	1 2 3 4 5 6 control	20 20 20 20 20 20 20	16 14 13 10 11 14 19	80 70 65 50 55 70 95	15.8 26.3 31.6 47.4 42.1 26.3 ——

†Relative percent survival.

And the *r* value of RPS and antibody showed that the protect of vaccine is moderately linear correlation with antibody in mucus, while low linear correlation with antibody in serum (**Table 5**). Interestingly, the value of *r* <0.05 between antibody in mucus and that in serum showed no correlation directly, which verified the relative independence of mucosal immunity from system immunity after immersion vaccination of grouper.

**Table 5:** The Pearson correlation coefficient(*r*) of RPS and antibody titer of immunized grouper with inactive *V. harveyi* vaccine.

	RPS (%)	Antibody in serum	Antibody in mucus
RPS (%)	1		
Antibody in serum	0.320028265	1	
Antibody in mucus	0.728643536	0.041561845	1

## Discussion

Many studies on immersion vaccination in fish have been reported in recent years (1–3), However, injection immunization technology is still the mainstream of application in the worldwide application (2, 3, 8, 20, 25, 26), whether manual injection or mechanical injection (25, 26). because immersion vaccination has numerous advantages over the injection methods including minimal pain for fish, lower labor costs and time coupled with greater operator safety especially for large numbers of small fish (2, 3, 18). However, immersion vaccination also possesses drawbacks such as the generation of weak immune responses and a large amount of vaccine is necessary and this can increase costs (2, 26). So there has been progress in defining uptake, processing, presentation and reaction of antigens in aquatic animal mucosal immunity during immersion vaccination, just to enhance immunogenicity and promote or regulate the level or type of immune response. We found that the inactivated *V. harveyi* vaccine induced a peak mucosal antibody titer earlier than that for serum indicating a more robust mucosal immunity in response to immersion vaccination. Consistent with these observations, tissue expression of IgM mRNA in the gill and skin mucosa peaked 4 days following vaccination and then declined. Interestingly, both fish showed similar patterns and this was consistent with the role these tissues play in the early stages of resisting pathogen invasion (19).

It was generally recognized that fish mucosal immunity exists and is relatively independent of the whole body immune system (3, 5, 8, 9, 18, 19). But most of them are discussed from the aspects of reaction process and reaction intensity. This paper introduces Parson Correlation Coefficient analysis for the first time, and verifies the relative independence of mucosal immunity objectively and mathematically. The value of *r* was so small between antibody in mucus and that in serum, which showed no correlation directly, and meant the relative independence of mucosal immunity from system immunity after immersion vaccination.

### Changes in Gills and Skins After Immersion

Mucosal-related tissues are key elements in preventing pathogen entry (8, 9). The mucosal lymphoid tissues (MALT) include nasopharynx-associated lymphoid tissues (NALT), gill-associated lymphoid tissues (GIALT), skin-associated lymphoid tissues (SALT) and gut-associated lymphoid tissues (GALT) (3). The particular systems responsible for immunity resulting from immersion vaccination are currently unclear. There are most likely also species differences for immune responsiveness (9, 10). There are many species of fish, such as seabream (*Sparus aurata*) *(* 6), Atlantic salmon(*Salmo salar*) *(* 27), IgM presence in the skin mucus was signifificantly lower than in the serum, while no clear differences were detected between skin mucus and serum Ig of carp (19) and olive flounder (28). In our study, the titer level of Ig in skin mucus was same as that in serum, but IgM and MHC II expression levels in the gills were much higher than that in the skin and hindgut indicating the gills for both fish species were robust mucosal immune response organs.

Of course, immersion inoculation utilizes mucosal immunity while parenteral administration of antigen induces a systemic reaction and specific antibody production (10, 29), so gill, skin and hindgut show different immune responses after immersion vaccination. It is reported that the soluble extracellular products of pathogenic *V. harveyi* stains showed strong toxicity to crucian carp and the fibroblasts of grass carp (16), so there are soluble and insoluble antigens both in the inactivated *V. harveyi* vaccine. The skin serves as the major organ for antigen uptake and particles remain in both skin and gill tissues for at least 24 days following exposure and a minority are transported to the spleen and kidney (30). And soluble bovine serum albumin (BSA) and insoluble fluorescent latex microspheres were both phagocytosed *via* the epithelium in channel catfish and adsorption of particulates has been documented in the skin and gill tissues of rainbow trout after immersion vaccination (6, 31). Hence, there are some differences in MHC II expression between gill and skin, but the *r* of the two organs IgM expression is almost 1.

The gill mucosal and immune-related tissues contain both macrophages and cells capable of expressing MHC (25, 32, 33), This has been confirmed in ultrastructural analysis of the gills of *Oncorhynchus tschawytscha* (31) and IgM-producing cells have also been detected in gill of *Siniperca chuatsi* (34), and some cells that most likely are dendritic cells were found in Ultrastructure of gills (32), which can be considered to be the main antigen presenting cells (35). We found that IgM gene expression levels in the gills were significantly increased following immunization and were much greater than for the skin mucosa for the grouper and crucian carp. This indicated that the gills were the primary local immune response organs that mounted a local response to the immersion antigen.

### Hypothesis of the Immune Synergy of the Hindgut-Liver-Spleen

IgM mRNA levels were also higher in spleen and HK following vaccination demonstrating the importance of systemic immunity. Previous studies have demonstrated that IgM mRNA in spleen, HK, gills and hindgut of *S. chuatsi* increased after intraperitoneal injection of inactivated *Flavobacterium columniformis* (33). Rainbow trout immersion inoculation with *Yersinia rugosa* increased IgM mRNA levels in the HK but not in the rear kidney (36). The latter was consistent with higher levels of plasma cells in the front kidney and our experimental results were consistent with these studies.

The expression of IgM mRNA in the hindgut was also higher than that for the skin mucosa in the present study. IgM production in most teleost intestine has not been reported for the Peyer’s plaques, M cells, sIgA, and J chains (1, 7, 8, 19, 37). In sheepshead (*Archosargus probatocephalus*), a noncovalent dimer of covalent dimeric subunits has been described associated to a 95-kDa secretory component like protein (7, 38), but this was not found in bile of the same species and also never observed in other teleost (8). In addition, there is no evidence of specific homing of mucosal lymphocytes to the fish intestine (37). In our study, IgM mRNA expression in the intestine after immersion was generally higher than skin. This was most likely the result of antigen absorption by the digestive tract during immersion and links systemic and local mucosal immunity (3). However, organ IgM mRNA expression levels changed with time and the hindgut, liver and spleen followed the same temporal trend. Parenthetical administration induces systemic reactions and the production of specific antibodies. In fish, bile can also provide immune effector and may cooperate with the intestinal tract and liver while maintaining an internal balance and is a unique mechanism of immunity (39). For instance, molecular adaptation mechanisms involved in fish intestine-liver immunity is related to soybean meal stress (40). However, histochemical analysis has shown that the numbers of lymphocytes in liver tissues was relatively small but abundant in the vascular system (41). It is reported that the artery supplying blood to the spleen originates from the gastric arteries of the dorsal aorta in the *Chiloscyllium punctatum (* 42),and 4 days after fertilization, Hox11 gene expression in a small area of the gut of the zebrafish embryo indicates the appearance of the splenic primordium (43), showed the possibility of close immunity connection between spleen and hindgut. The spleen and HK are the primary lymphoid organs of fish and are the primary sites of antigen capture (3, 26). Serum antibodies originate in HK and plasma cells of the spleen and kidney are distributed around blood vessels (32, 34). For instance, the toxic effects of microcystins to zebrafish were reflected in the spleen, intestine and gills with the up-regulation of immune-related genes that demonstrates the relationship between spleen and mucosal immunity (41).

On the contrary, the *r* value of the HK is relatively low with other organs(| *r* | < 0.4, means low linear correlation), except for the slightly higher correlation with spleen (0.5 ≤ | *r* | < 0.8, means moderately linear correlation). The HK and spleen are the major sites for trapping of antigens and the production of antibodies, and the predominant source of serum Ig is suggested to be plasma cells in the HK (44, 45), making this teleost tissue analogous to the bone marrow of mammals (46).As this is the case, exogenous antigen presentation in HK would be anticipated (47). In this study, the peak expression of IgM and MHCII in grouper HK were 21^st^ day both, which were later than 14^th^ day in spleen, showing that the response to antigen in HK is slightly later than that in spleen. And the peak of blood antibody titer is also 21^st^ day, which verified that the main source of serum Ig is the plasma cells in HK (44, 45).

Together these data indicated that the immunity provided by the hindgut-liver-spleen system is profoundly important for fish but the specific mechanisms needs further tissue and molecular confirmations for a complete explanation of this cellular pathway.

### Comparison of IgM Gene Expression Changes Between the Grouper and Crucian Carp

Many studies believe that tilapia, crucian carp may be used as a substitute for the valuable mariculture animals in relevant experimental research (16, 17, 48, 49). In our experiments, *V. harveyi* strain can infect Crucian carp and grouper both, and the LD_50_ of the strain to crucian carp and group are at same level calculated by R-M method. We also examined whether there were differences in the immune responses between the grouper and crucian carp by challenging with live *V. harveyi* cells. The RPS levels were basically the same for both fish. They both peaked in the 4^th^ week, remained at their peak in the 5^th^ week, and dropped to the level of the 2^nd^ week in the 6^th^ week, just the grouper RPS level is somewhat higher than the carp (47.4% vs. 44.4%).

However, they were not consistent that the internal immune response between that of crucian carp and grouper after immersion the *Vibrio* vaccine. IgM expression levels in the grouper spleen was up-regulated on day 3 following immunization and peaked on day 14^th^ while in crucian carp levels were maximal on day 7^th^. In spite of this, IgM expression levels for both fish were high from day 7^th^ to 21^st^. The expression levels in HK increased significantly later and were maximal at day 21^st^ and these expression levels for the grouper and crucian carp were significant with increases of 40.6- and 28.3-fold, respectively. Therefore, IgM levels in the HKs of these fish were consistent. In contrast, IgM expression levels in the hindgut peaked for the grouper at day 14^th^ but on day 4^th^ in the crucian carp. Previous studies have indicated that Ig-positive cells in the sea bass, salmon and cyprinid fish are distributed in their intestinal mucosa and levels differed between species (3, 24, 45, 50). This difference may be due to differences between species, which have also been found in marine fish after both immersion and injection vaccination protocols (20).

Therefore, different species fish cannot be substituted for each other in immune evaluation, especially those with distant evolutionary relatives, whose immune protection phenomena may be similar, but their internal immune protection patterns are quite different.

## Data Availability Statement

The original contributions presented in the study are included in the article/**Supplementary Material**. Further inquiries can be directed to the corresponding authors.

## Ethics Statement

The animal study was reviewed and approved by The Animal Experiment Committee of Pearl River Fisheries Research Institute, Chinese Academy of Fisheries Sciences.

## Author Contributions

HG and ZH conceived the projects. HG, QW, YL, CZ, CS, and JT performed the experiments. HG, QW, ZC, JT and ZH did data analysis. HG and QW wrote the manuscript. QW and JT revised the manuscript. All authors contributed to the article and approved the submitted version.

## Funding

The work was funded by grants from National Key Research and Development Program of China (2019YFD0900103); Central Public-interest Scientific Institution Basal Research Fund, CAFS (2020TD45); Guangzhou Science and Technology Plan Project (201904020004).

## Conflict of Interest

The authors declare that the research was conducted in the absence of any commercial or financial relationships that could be construed as a potential conflict of interest.

## Supplementary Material

Supplementary Figure 1 Agarose gel electrophoresis of total RNA isolated from fish tissues. Lanes 1 to 4. *Carassius auratus* and Lanes 5 to 8. *Epinephelus coioides* including samples from HK, spleen, gills and skin, respectively. Due to the large number of RNA samples, we only show the total RNA in several tissues extracted at a time point.

Supplementary Figure 2 Melting curves of β-actin (left, Tm: 92 ± 0°C) and IgM (right, Tm: 87 ± 0°C). The fusion curve analysis showed that the PCR products of β-Actin and IgM genes showed a sharp single peak, and there are no other stray peaks.
