# Supplementary figures and images for: Study on Immune Response of Organs of Epinephelus coioides and Carassius auratus After Immersion Vaccination With Inactivated Vibrio harveyi Vaccine
Source: Front Immunol. 2021 Feb 9;11:622387. doi: 10.3389/fimmu.2020.622387 (PMC7900426; doi:10.3389/fimmu.2020.622387)

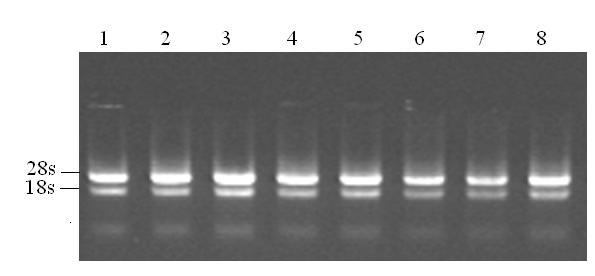

Supplement: Supplementary Figure 1 — Agarose gel electrophoresis of total RNA isolated from fish tissues. Lanes 1 to 4. Carassius auratus and Lanes 5 to 8. Epinephelus coioides including samples from HK, spleen, gills and skin, respectively. Due to the large number of RNA samples, we only show the total RNA in several tissues extracted at a time point. [file DataSheet_1.zip › Supplementary Figure 1.tif]

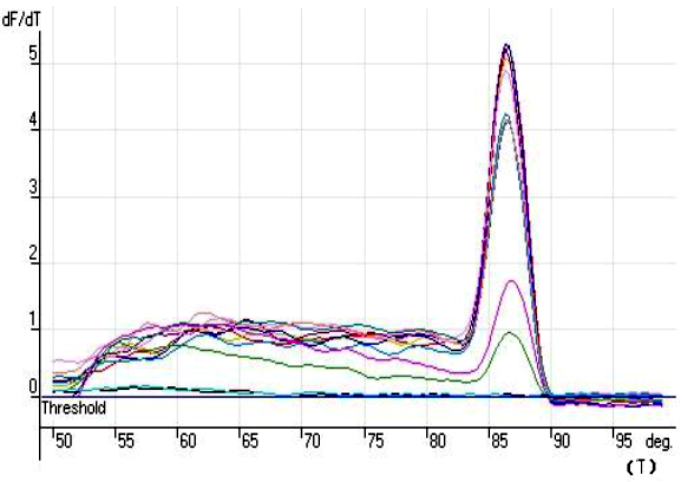

Supplement: Supplementary Figure 1 — Agarose gel electrophoresis of total RNA isolated from fish tissues. Lanes 1 to 4. Carassius auratus and Lanes 5 to 8. Epinephelus coioides including samples from HK, spleen, gills and skin, respectively. Due to the large number of RNA samples, we only show the total RNA in several tissues extracted at a time point. [file DataSheet_1.zip › Supplementary Figure 2A.tif]

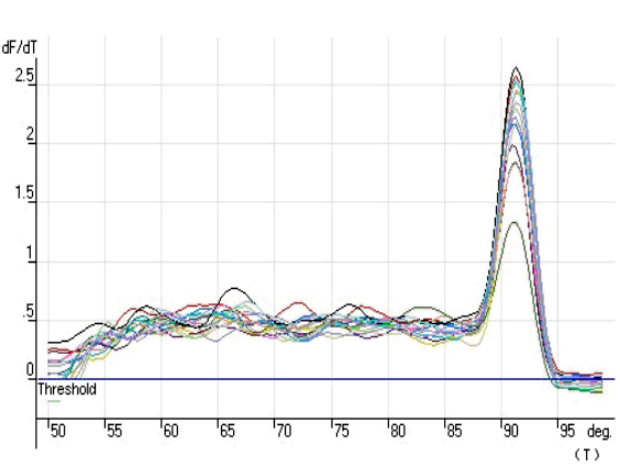

Supplement: Supplementary Figure 1 — Agarose gel electrophoresis of total RNA isolated from fish tissues. Lanes 1 to 4. Carassius auratus and Lanes 5 to 8. Epinephelus coioides including samples from HK, spleen, gills and skin, respectively. Due to the large number of RNA samples, we only show the total RNA in several tissues extracted at a time point. [file DataSheet_1.zip › Supplementary Figure 2B.tif]
